# Elevated serum beta-2 microglobulin level predicts short-term poor prognosis of patients with *de novo* acute omicron variant COVID-19 infection

**DOI:** 10.3389/fcimb.2023.1204326

**Published:** 2023-07-13

**Authors:** Shengping Gong, Ruishuang Ma, Ting Zhu, Xiaoqin Ge, Rongrong Xie, Qingsong Tao, Cong Shi

**Affiliations:** ^1^ Cancer Radiotherapy and Chemotherapy Center, the First Affiliated Hospital of Ningbo University, Ningbo, Zhejiang, China; ^2^ Laboratory of Stem Cell Transplantation, the First Affiliated Hospital of Ningbo University, Ningbo, Zhejiang, China; ^3^ Key Laboratory of Precision Medicine for Atherosclerotic Diseases of Zhejiang Province, the First Affiliated Hospital of Ningbo University, Ningbo, Zhejiang, China

**Keywords:** omicron, COVID-19, short-term, prognosis, β2-MG

## Abstract

**Background:**

The devastating coronavirus disease of 2019 (COVID-2019) epidemic has been declared a public health emergency, resulting in a worldwide pandemic. The omicron variety is the most common epidemic mutant strain in the globe. Serum beta-2 microglobulin (β2-MG) is associated with endothelial cell injury and has value in monitoring the progression of inflammation in infected individuals. Nonetheless, the potential functions of β2-MG in omicron remain elusive.

**Methods:**

To investigate the prognostic value of serum β2-MG levels at diagnosis, we retrospectively analyzed a cohort of 240 people with omicron. Over the course of 65 days, all patients were monitored, and death was the primary outcome. Patients were allocated to two groups: those with high and low β2-MG levels. The Kaplan–Meier method was used to examine OS, and the log-rank test was used to compare them. Univariate and multivariate Cox hazard models were used to determine the prognostic significance.

**Results:**

Our results revealed that β2-MG was significantly elevated in omicron. β2-MG levels in severe patients were higher than in mild-to-moderate patients, and the difference was statistically significant. Timely, interleukin-6 (IL-6) and interleukin-10 (IL-10) were observed to be significantly increased in individuals exhibiting elevated levels of β2-MG. In addition, patients exhibiting elevated levels of β2-MG demonstrated a statistically significant decrease in overall survival (OS, *P* < 0.0001). An elevated β2-MG level (≥4.72 mg/l) was found to be an independent, adverse prognostic factor for OS in omicron patients, according to multivariate Cox proportional hazards regression analysis (*P* = 0.001).

**Conclusion:**

Serum β2-MG level at initial diagnosis was significantly correlated with omicron severity and prognosis. Thus, we propose that β2-MG may be an independent poor additional prognostic factor in patients with omicron.

## Background

Coronavirus disease of 2019 (COVID-19) is a widely recognized disease attributed to the pathogenic agent, the coronavirus type 2, associated with severe acute respiratory syndrome (SARS-Cov-2). Throughout the transmission process, the unique coronavirus developed and mutated, forming many mutant strains, including five “variants of concern,” Alpha, Beta, Gama, Delta, and Omicron. At the moment, the omicron variety is the most common pandemic strain in the globe. Infection with the omicron variation might rapidly escalate the systemic inflammatory response, aberrant coagulation function, multiorgan function damage, and other pathophysiological alterations, implying that serious cases should be treated differently than other COVID-19 strains. Ningbo experienced the epidemic of the omicron BA.5 and BF.7 between December 2022 and January 2023 in China. The medical facilities established specialized centers for treating COVID-19, specifically targeting the omicron variant of the virus. The patients were treated in a systematic and organized way.

Omicron infection caused a series of immune responses, including cellular and humoral immunity. A number of risk factors have been identified in association with the progression of severe COVID-19 disease, such as lymphocyte subpopulation, interleukin (IL), and C-reactive protein (CRP) (Akbari et al., 2020; Liu et al., 2020; Liu et al., 2020).

Beta-2 microglobulin, also known as β2-MG, is classified as a low molecular weight protein. β2-MG is a polypeptide chain that forms a constituent of the human leukocyte antigens (HLAs). It is released into the bloodstream subsequent to dissociation from HLA as a result of the regular metabolic processes that occur within the body (Ma et al., 2012). Most β2-MG is made by epithelial, mesenchymal, and lymphocytes, but all nucleated cells in the human body contribute to its production. In physiological conditions, the serum β2-MG level is low. The measurement of serum β2-MG level is regarded as a crucial prognostic factor for survival and indicates the extent of tumor burden, independent of other variables (Min et al., 2002). Chronic renal failure, dialysis patients, proliferative lymphatic diseases, inflammation, infection, and other conditions are associated with elevated β2-MG levels (Wu et al., 2017). Elevated serum β2-MG levels were found in 34 patients with COVID-19 infection and were associated with poor prognosis (Conca et al., 2021). Whether β2-MG is an independent prognostic factor is unclear due to the limited sample size. Henceforth, a retrospective analysis was conducted on the serum β2-MG levels during diagnosis to ascertain its significant prognostic worth in individuals with omicron infection.

## Materials and methods

### Patients

The study had 240 patients who were diagnosed with the omicron variant of COVID-19 through laboratory testing. Of these patients,152 were men and 88 were women, with an average age of 76 years (between 32 and 100). The patients received centralized treatment at the First Affiliated Hospital of Ningbo University between 29/12/2022 and 15/01/2023. The study involved a follow-up period of 65 days for all patients, with the primary outcome defined as death. The confirmation of SARS-CoV-2 infection among all patients was established through virus nucleic acid testing, which necessitated the presence of positive outcomes in SARS-CoV-2 RNA. Peripheral blood samples from 200 healthy donors who had no infectious disease were the control group from the First Affiliated Hospital of Ningbo University health management center. The primary outcome of the study was in-hospital mortality. In the cohort, 63 died whereas 177 recovered and were discharged from the hospital until 18/01/2023. The study participants were categorized based on the severity of their illness, either mild-to-moderate or severe, in accordance with the criteria outlined in the “Diagnosis and Treatment Protocol for COVID-19 Patients (tentative 8th edition)” (Wang et al., 2021).

The Ethics Committee of the First Affiliated Hospital of Ningbo University approved the retrospective review of the aforementioned records in accordance with the principles outlined in the Declaration of Helsinki. Prior to their participation in the study, all adult subjects provided informed consent.

### Serum β2-MG determination

The sample of the blood was collected from the peripheral veins of the participants following a period of strict fasting lasting no less than 6 h. The turbidimetric immunoassay method was utilized to measure the level of serum β2-MG. The reagents underwent utilizing Beckman’s β2-MG kit in accordance with their instructions, utilizing an automated biochemical analyzer (Beckman Dxl 800).

### Absolute count of lymphocyte subsets and cytokine detection

Peripheral blood samples collected from all subjects were anticoagulated using EDTA. The enumeration of lymphocyte subsets and cytokines was performed using a BD FACSCanto flow cytometer to obtain absolute counts (BD, USA). The enumeration of lymphocyte subsets was performed using the BD Multitest 6-Color TBNK reagent, whereas the identification of cytokines was carried out using the BD human Th1/Th2 cytokine kit. The experiments were conducted in accordance with the instructions provided in the product manual.

### Statistical analysis

SPSS 26.0 and GraphPad Prism 8.0 were employed for the statistical analysis. OS was calculated from the hospital diagnosis of omicron to 18/01/2023. OS was compared using the log-rank test and the Kaplan–Meier method of analysis. Cox proportional hazard regression was used to do multivariable analysis. The statistical tests that can be used are either Student t-test or Mann–Whitney *U* test. The *U* test was employed to evaluate disparities in the dispersion of continuous variables among various categories, whereas for categorical data, chi-squared tests were used. The X-Tile software was used to determine the cutoff point of β2-MG (Camp et al., 2004). The determination of the optimal threshold for differences in survival was predicated on the attainment of the most minimal *P*-value *via* the employment of the log-rank test, which was determined to be 4.72 mg/l. Receiver operating characteristic (ROC) curve analysis was used to determine β2-MG’s usefulness as a diagnostic tool. The statistical significance of the result was established based on *P* < 0.05.

## Results

### Patient characteristics

The data of 240 omicron variant COVID-19 patients, including 152 men and 88 women, were followed up for 65 days. The median OS of these patients was 44 (range 2–63) days, and 63 of them were fatal. The study included a total of 240 patients, of which 121 were classified as mild-to-moderate and 119 were classified as severe. The mild-to-moderate group consisted of 51 women and 70 men with an average age of 73 years (range: 32–100). The severe group consisted of 82 men and 37 women, with a median age of 78 years (range: 34–100). According to the clinical guidelines, all patients were categorized as 121 mild-to-moderate and 119 severe patients. Detailed information is provided in **Table 1**.

**Table 1:** Demographics and clinical characteristics of patients with omicron.

Variable	Condition on admission				Outcome			
	Mild-to-moderate patients(*n*=121)	Severe patients (*n*=119)	Statistics	*P value*	Survivor (*n*=177)	Non-survivor (*n*=63)	Statistics	*P value*
Male/female, n	70/51	82/37	χ^2 = ^3.158	0.076	109/68	43/21	χ2 = 0.634	0.426
Age **[**years, median (quartile)**]**	73 (66∼84)	78 (70∼84)	Z =−2.063	0.039	75 (67.5∼84)	77 (69∼83)	Z =−1.247	0.212
Comorbidity, n								
Hypertension	72/49	75/44	χ^2 = ^0.313	0.576	107/70	40/23	χ2 = 0.181	0.671
Diabetes	30/91	38/81	χ^2 = ^1.506	0.220	47/130	21/42	χ2 = 1.052	0.305
Cardiovascular disease	10/111	3/116	χ^2 = ^3.863	0.084	10/167	3/60	χ2 = 0.071	1.000
Cerebrovascular disease	5/116	5/114	χ^2 = ^0.001	1.000	6/171	4/59	χ2 = 1.019	0.295
Pulmonary disease	3/118	4/115	χ^2 = ^0.165	0.721	5/172	2/61	χ2 = 0.020	1.000
Chronic kidney disease	2/119	1/118	χ^2 = ^0.321	1.000	3/174	0/63	χ2 = 1.081	0.569
Malignant tumor	6/115	7/112	χ^2 = ^0.100	0.752	9/168	4/59	χ2 = 0.145	0.748
Laboratory parameters								
WBC [×10^9^/L, median(quartile)]	6.2 (4.35∼9.7)	7.6 (5.0∼13)	Z =−2.654	0.008	6.9 (4.4∼10.3)	7.2 (5.2∼13.3)	Z =−1.739	0.082
NE [×10^9^/L, median(quartile)]	4.9 (3.1∼8.05)	6.2 (3.8∼11.2)	Z =−3.053	0.002	5.3 (3.2∼9.0)	6.3 (4.2∼11.6)	Z =−2.035	0.042
ALC [×10^9^/L, median(quartile)]	0.7 (0.5∼1.2)	0.5 (0.4∼0.8)	Z =−3.318	0.001	0.7 (0.5∼1.1)	0.5 (0.3∼0.7)	Z =−3.908	<0.0001
RBC [×10^9^/L, median(quartile)]	3.9 (3.4∼4.3)	3.9 (3.4∼4.4)	Z =−0.053	0.958	3.9 (3.4∼4.3)	3.9 (3.2∼4.4)	Z =−0.446	0.656
HB [g/L, median (quartile)]	121 (107∼131)	119 (106∼138)	Z =−0.200	0.842	121 (108∼133)	119 (99∼140)	Z =−0.371	0.711
PLT [×10^9^/L, median (quartile)]	182 (136.5∼241.5)	192 (140∼252)	Z =−0.587	0.557	191 (143.5∼249)	155 (123∼238)	Z =−2.143	0.032
CRP [mg/L, median (quartile)]	31.6 (12.9∼61.6)	51.2 (28.1∼111.9)	Z =−4.092	<0.0001	38.4 (16.4∼68.4)	63.7 (24.6∼114.8)	Z =−3.192	<0.0001
β2-MG [mg/L, median (quartile)]	3.1 (2.2∼4.4)	4.5 (2.6∼7.3)	Z =−3.689	<0.0001	3.12 (2.23∼4.58)	5.87 (3.1∼12.1)	Z =−5.685	<0.0001
Lymphocyte subpopulation								
CD3 absolute value [/μL, median (quartile)]	322 (206.5∼627)	207 (151∼355)	Z =−3.148	0.002	291 (190∼575)	197 (131.5∼318)	Z =−2.601	0.009
CD4 absolute value [/μL, median (quartile)]	176 (108∼320.5)	112 (77∼217)	Z =−2.609	0.009	156 (95∼295)	113 (76∼226.5)	Z =−1.433	0.152
CD8 absolute value [/μL, median (quartile)]	126 (67.5∼246.5)	70 (37∼140)	Z =−2.904	0.004	118 (59∼231)	67 (31∼108)	Z =−3.201	0.001
NK absolute value [/μL, median (quartile)]	130 (72.5∼209)	90 (46∼151)	Z =−2.427	0.015	112 (71∼208)	80 (31∼150.5)	Z =−2.562	0.010
CD19 absolute value [/μL, median (quartile)]	73 (32.5∼130.5)	64 (36∼110)	Z =−0.328	0.743	72 (36∼128)	66 (35∼12.1)	Z =−0.608	0.543
Cytokines								
IL-6 [pg/mL, median (quartile)]	14.5 (9.6∼29.8)	40.2 (15.5∼95.8)	Z =−4.762	<0.0001	18.2 (10.1∼52.3)	45.3 (18.9∼137.1)	Z =−4.466	<0.0001
IL-10 [pg/mL, median (quartile)]	7.9 (5.4∼12.9)	12.4 (8.2∼24)	Z =−4.641	<0.0001	9.5 (6.5∼15.3)	14 (7∼43.1)	Z =−2.994	0.003

WBC, white blood count; NE, neutrophil; ALC, absolute lymphocyte count; RBC, red blood count; HB, hemoglobin; CRP, C reactive protein; β2-MG, beta-2 microglobulin; IL-6, interleukin-6; IL-10, interleukin-10.

### X-Tile and ROC curve analyses

**Figure 1:**
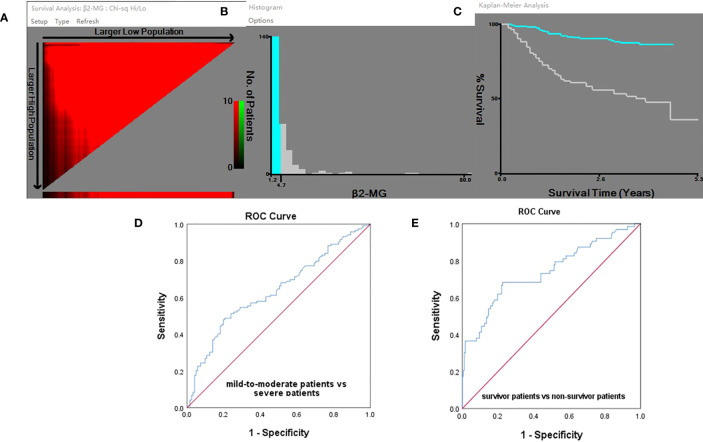
**(A-E)** X-Tile analyses and ROC curve. **(A)** X-Tile plot of the training sets, with the plot of matched validation sets shown in the smaller inset. The optimal cutoff points highlighted by the red circle in A is shown on the histograms of the entire cohort in **(B)** and Kaplan–Meier plot **(C)**. According to the X-Tile program, the optimum cutoff points for β2-MG was determined to be 4.72 mg/l. The ROC analysis provided a cutoff value of 4.72 mg/l for predicting COVID-19 severity **(D)** and mortality **(E)** using β2-MG.

The X-Tile algorithm established the ideal threshold for changes in survival based on the achievement of the most minimal *P*-value *via* the log-rank test, which was determined to be 4.72 mg/l (**Figures 1A–C**). Additionally, receiver operator characteristic (ROC) analysis identified a cutoff value of 4.72 mg/l for β2-MG to predict severity and mortality in omicron variant COVID-19 cases, with an area under the ROC curve of 0.638 (95% confidence intervals 0.568–0.708, *P* < 0.0001; **Figure 1D**) and with an area under the ROC curve of 0.741 (95% confidence intervals 0.664–0.819, *P* < 0.0001; **Figure 1E**), respectively. The high β2-MG level group had a β2-MG level of >4.72 mg/l, whereas the low β2-MG level group had a β2-MG level of ≤4.72 mg/l.

### Serum β2-MG level in peripheral blood of omicron patients

**Figure 2:**
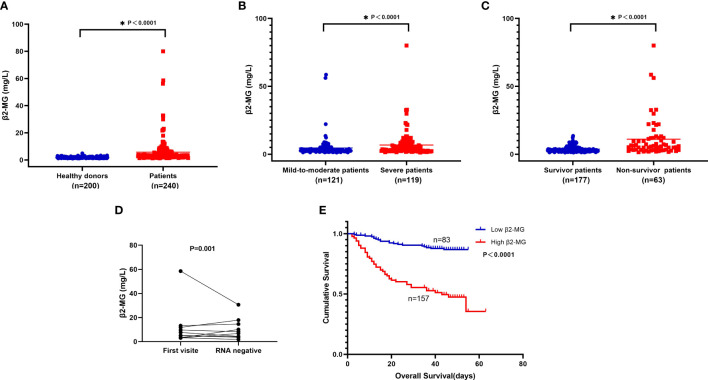
**(A-E)**. **(A)** Compare serum β2-MG between 200 healthy donors and 240 patients. **(B)** Compare serum β2-MG between 121 mild-to-moderate patients and 119 severe patients. **(C)** Compare serum β2-MG between 177 survivor patients and 63 non-survivor patients. **(D)** The dynamic changes of serum β2-MG in omicron patients whose viral nucleic acid test turned negative. **(E)** Overall survival according to serum β2-MG in omicron patients.

In our cohort, significant elevation was observed in the serum β2-MG in omicron variant COVID-19-infected individuals as compared with the one without COVID-19 infection. The β2-MG level median among individuals diagnosed with the omicron variant was observed to be higher than that of 200 healthy donors (3.44 vs. 1.73 mg/l, *P* < 0.0001; **Figure 2A**). Severe patients showed significantly elevated serum β2-MG compared with mild-to-moderate patients. The β2-MG level of non-surviving patients exhibited a higher increase in comparison with that of surviving patients (*P* < 0.05) (**Figures 2B, C**).

### The relationship between serum β2-MG level and other factors in clinics and laboratory

The omicron variant represents a recently discovered variant of the SARA-CoV-2 virus. The study involved categorizing COVID-19 patients into two distinct groups to examine the potential correlation between the β2-MG level and various clinical and laboratory characteristics. The findings indicate that the high β2-MG group exhibited advanced age and significantly elevated levels of CRP (*P* = 0.033), IL-6 (*P* = 0.001), and IL-10 in comparison with the low β2-MG group (*P* = 0.023). No significant variations were observed in other factors between the two groups (**Table 2**).

**Table 2:** Comparison between omicron with low β2-MG group and high β2-MG group in 240 patients.

Variable	Low β2-MG group (*n*=157)	High β2-MG group (n=83)	Statistics	*P value*
Male/female, n	96/61	56/27	χ^2 = ^0.935	0.334
Age **[**years, median (quartile)**]**	73 (67∼80)	81 (70∼87)	Z =-3.828	<0.0001
Comorbidity, n				
Hypertension	89/68	58/25	χ^2 = ^3.981	0.046
Diabetes	41/116	27/56	χ^2 = ^1.101	0.294
Cardiovascular disease	9/148	4/79	χ^2 = ^0.088	0.512
Cerebrovascular disease	4/153	6/77	χ^2 = ^2.980	0.086
Pulmonary disease	6/151	1/82	χ^2 = ^1.313	0.237
Chronic kidney disease	3/154	0/83	χ^2 = ^1.606	0.553
Malignant tumor	8/149	5/78	χ^2 = ^0.091	0.770
**Vaccination, n**	71/86	34/49	χ^2 = ^0.400	0.572
Laboratory parameters				
WBC [×10^9^/L, median (quartile)]	7.2 (4.6∼10.5)	7.1 (4.7∼10.7)	Z =−0.095	0.924
NE [×10^9^/L, median (quartile)]	5.7 (3.4∼9.5)	5.7 (3.2∼9.0)	Z =−0.236	0.814
ALC [×10^9^/L, median (quartile)]	0.6 (0.4∼1.1)	0.6 (0.4∼0.9)	Z =−1.341	0.180
RBC [×10^9^/L, median (quartile)]	3.9 (3.4∼4.3)	3.8 (3.2∼4.4)	Z =−1.407	0.159
HB [g/L, median (quartile)]	122 (109∼135)	118 (104∼131)	Z =−1.492	0.136
PLT [×10^9^/L, median (quartile)]	192 (141.5∼255.5)	172 (130∼220)	Z =−1.789	0.074
C reactive protein [mg/L, median (quartile)]	40 (19.5∼81.3)	50.7 (20.4∼114.8)	Z =−2.126	0.033
β2-MG [mg/L, median (quartile)]	2.7 (2.1∼3.4)	7.3 (5.2∼10.1)	Z =−12.736	<0.0001
Lymphocyte subpopulation				
CD3 absolute value [/μL, median (quartile)]	271 (161.8∼533.5)	241.5 (168.5∼497)	Z =−0.348	0.728
CD4 absolute value [/μL, median (quartile)]	141 (86.8∼278.3)	157 (88.8∼235.5)	Z =−0.184	0.854
CD8 absolute value [/μL, median (quartile)]	101.5 (58∼201.8)	88 (37.8∼249)	Z =−0.648	0.517
NK absolute value [/μL, median (quartile)]	105 (68∼177.3)	120.5 (52.3∼207.3)	Z =−0.072	0.942
CD19 absolute value [/μL, median (quartile)]	70.5 (33.3∼126.5)	76 (41.8∼110.5)	Z =−0.276	0.782
Cytokines				
IL-6 [pg/mL, median (quartile)]	18.4 (10.4∼51.4)	41.5 (15.6∼120)	Z =−3.182	0.001
IL-10 [pg/mL, median (quartile)]	9.6 (6.3∼15.6)	11.5 (7.2∼28.7)	Z =−2.281	0.023

WBC, white blood count; NE, neutrophil; ALC, absolute lymphocyte count; RBC, red blood count; HB, hemoglobin; CRP, C reactive protein; β2-MG, beta-2 microglobulin; IL-6, interleukin-6; IL-10, interleukin-10.

### Changes in the levels of β2-MG in omicron patients during follow-up

A group of 10 individuals who were recently diagnosed with the omicron variant were monitored over a period of 2 weeks. Following the follow-up, the majority of patients whose virus nucleic acid test yielded negative results exhibited an improvement in their β2-MG levels (**Figure 2D**).

### High β2-MG level was associated with a poor prognosis

The group with high levels of β2-MG exhibited a significantly shorter median OS in comparison with the group with low levels of β2-MG (36 vs. 45 days, *P* < 0.0001; **Figure 2E**).

The univariate analysis revealed a negative association between the OS and higher CRP (*P* < 0.0001), higher IL-6 (*P* < 0.0001), higher IL-10 (*P* < 0.0001), higher WBC (*P* < 0.0001), higher NE (*P* = 0.001), higher β2-MG (*P* < 0.0001), lower levels of CD3 lymphocyte absolute count (*P* = 0.001), CD4 lymphocyte absolute count (*P* = 0.031), CD8 lymphocyte absolute count (*P* < 0.0001), CD4+CD8+ absolute lymphocyte count (*P* = 0.028), NK lymphocyte absolute count (*P* = 0.003), CD19 lymphocyte absolute count (*P* = 0.039), lower ALC (*P* = 0.001), lower HB (*P* = 0.015), and lower PLT (*P* = 0.007).

Multivariate analyses showed that lower NK lymphocyte absolute count (*P* = 0.042) and higher β2-MG (*P* = 0.001) were independent adverse factors for worse OS (**Table 3**).

**Table 3:** Univariate and multivariate analyses of different prognostic parameters for overall survival of 240 patients with omicron.

Variables	Univariate analysis for OS		Multivariate analysis for OS		
	*P-value*	95% CI	*P-value*	*HR*	95% CI
Age ≥ 60 (years)	0.272	40.047–67.953	–	–	–
Gender (male)	0.346	46.910–53.121	–	–	–
CRP ≥ 53.99 mg/l	<0.0001	42.826–65.174	0.880	1.062	0.488–2.312
IL-6 ≥ 38.7 pg/ml	<0.0001	42.890–65.110	0.359	1.558	0.605–4.014
IL-10 ≥ 29 pg/ml	<0.0001	42.890–65.110	0.317	1.581	0.645–3.879
CD3# < 236/μl	0.001	34.048–43.163	0.655	0.741	0.198–2.767
CD4# < 106/μl	0.031	35.189–45.159	0.328	1.586	0.629–3.995
CD8# < 115/μl	<0.0001	34.697–42.938	0.077	0.291	0.074–1.141
CD4+CD8+# < 3	0.028	37.559–44.888	0.537	0.742	0.288–1.914
NK# < 90/μl	0.003	33.970–43.594	0.042	0.466	0.223–0.974
CD19# < 118/μl	0.039	38.418–45.044	0.451	0.610	0.169–2.205
WBC ≥ 14.91×10^9^/l	<0.0001	31.066–56.934	0.089	0.121	0.011–1.379
NE ≥ 13.1×10^9^/l	0.001	26.900–61.100	0.541	2.122	0.190–23.691
ALC < 0.7×10^9^/l	0.001	37.703–44.616	0.650	0.771	0.250–2.373
RBC < 3.57×10^9^/l	0.082	28.905–79.095	0.584	1.302	0.507–3.346
HB < 100 g/dl	0.015	28.588–79.412	0.841	1.127	0.350–3.635
PLT < 134×10^9^/l	0.007	34.705–44.986	0.217	1.720	0.727–4.068
Hypertension	0.589	46.810–53.730	–	–	–
Cardiopathy	0.816	33.954–54.969	–	–	–
Diabetes	0.283	27.506–80.494	–	–	–
β2-MG ≥ 4.72 mg/l	<0.0001	29.090–56.910	0.001	3.822	1.775–8.227

CRP, C-reactive protein; IL-6, interleukin-6; IL-10, interleukin-10; WBC, white blood count; NE, neutrophil; ALC, absolute lymphocyte count; RBC, red blood count; HB, hemoglobin; PLT, platelet; β2-MG, beta-2 microglobulin.

## Discussion

The findings indicate that the level of β2-MG in individuals with omicron was elevated compared with those who were considered healthy. Furthermore, we showed that the pretherapy serum β2-MG levels were higher in severe patients than in mild-to-moderate patients and higher in death patients than in survivors. Additionally, serum β2-MG at a high level was associated with older age and higher CRP, higher IL-6, and higher IL-10. Serum β2-MG levels were found to be considerably higher in 10 newly diagnosed patients. It dropped dramatically after 2 weeks of treatment, and the virus nucleic acid test turned negative. Elevated serum β2-MG levels correlated with a shorter survival period in omicron patients, indicating that a higher serum β2-MG level reflects a poor prognosis in omicron patients. The results of the Cox regression analysis revealed that the increased levels of β2-MG were a significant independent prognostic factor for individuals diagnosed with omicron.

β2-MG is a protein present on the surface membrane of nearly all nucleated cells. It is notably prevalent in immune competent cells, specifically those that are activated, such as T and B lymphocytes and macrophages. High levels of β2-MG have been noted in cases of lymphoproliferative and rheumatic illnesses (Bethea and Forman, 1990). In addition, β2-MG could be used to track the progression of the illness in people with systemic lupus erythematosus (Font et al., 1986; Kim et al., 2010). The level of β2-MG in the blood can accurately reflect renal function and membrane renewal, and it has a relationship with tumor size and growth rate in diffuse large B-cell lymphoma (Kanemasa et al., 2017). β2-MG is a biomarker of acute kidney injury that is both early and sensitive (Wang et al., 2020). β2-MG is a structural protein that regulates immune recognition and immune recognition and immunoglobulin transport in the host (Montealegre et al., 2015; Wang et al., 2020). It can act directly on the cells to participate in the inflammatory response and regulate cytokine expression (Nomura et al., 2014). At the time when the body experiences an inflammatory response or metabolic abnormalities, the release of β2-MG increases, the degradation decreases, and the serum concentration of β2-MG elevates, particularly in severe patients (Li et al., 2014; Vianello et al., 2015). EBV, influenza, and CMV viruses are related to higher levels of β2-MG proteins (Cooper et al., 1984). Initial β2-MG levels as a predictor of the disease’s course and severity. Additionally, it has been suggested that β2-MG can tell the prognosis of patients with several tumor types, including colorectal cancer and Burkitt lymphoma (Kim et al., 2021; Na et al., 2021). The outcome is consistent with our research. In brief, β2-MG can partially capture the immune and inflammatory states, particularly in patients with hematological conditions and immune system disorders.

According to reports, individuals with considerably elevated levels of β2-MG typically had advanced disease and poor prognoses, whereas patients with low concentrations had better prognoses and longer survival times. In accordance with this study, higher β2-MG levels among omicron patients were among the ones with a higher risk of death and a shorter OS.

Our findings indicate that since the β2-MG is easy to determine and has a great consistency, β2-MG level measurements in routine clinical practice may help clinicians make treatment decisions and predict clinical outcomes. Our study has certain limitations. First, a retrospective study was done in this case, which is prone to selection bias and confounding factors. In addition, clinical data were insufficient, and some markers that might impact prognosis were left out of the study. Second, no comorbidities that might affect the result of omicron COVID-19 patients were included in the research. Third, the study did not investigate the processes that underpin the link between β2-MG and omicron COVID-19 severity and prognosis. Fourth, increased serum β2-MG levels are insufficient to indicate medical disease on their own. Hence, this study would benefit from a time-bound assessment of β2-MG elevation, specifically to determine how early postinfection β2-MG levels elevate. Nonetheless, compared with previous studies, the patient cohort in this study is homogenous, rendering the results reliable. It is worth noting that prospective cohort studies or randomized controlled trials may give more substantial evidence for this marker’s predictive significance.

## Conclusions

We found that elevated β2-MG in an individual was accompanied by elevated IL-6 levels and IL-10 levels, and increased β2-MG was linked with severe disease and poor prognosis in omicron patients. It can be convenient to assess the prognosis of omicron patients using serum β2-MG as an indicator of prognosis. These findings might aid in understanding the pathophysiology of omicron and establishing novel therapeutic strategies. The value of β2-MG should be evaluated in significant prospective trials because this study is a retrospective analysis and is only useful for generating a hypothesis.

## Data availability statement

The original contributions presented in the study are included in the article/supplementary material. Further inquiries can be directed to the corresponding authors.

## Ethics statement

All patients provided their informed written consent, which was recorded. The Ethics Committee of the First Affiliated Hospital of Ningbo University granted approval for project (2023028RS) and the project adhered to Helsinki to the principles outlined in its Declaration. The consent request was extended to encompass all co-authors to enable them to access to the data.

## Author contributions

SG collected and analyzed data and wrote the manuscript. CS analyzed data. CS and QT designed the research and reviewed the manuscript. RM, TZ, XG, and RX collected data. All authors read and approved the final manuscript.
